# The efficacy of mobile health in alleviating risk factors related to the occurrence and development of coronary heart disease: A systematic review and meta‐analysis

**DOI:** 10.1002/clc.23596

**Published:** 2021-03-16

**Authors:** Yue Xu, Hui Ye, Yuan Zhu, Shizheng Du, Guihua Xu, Qing Wang

**Affiliations:** ^1^ School of Nursing Nanjing university of Chinese Medicine Nanjing Jiangsu Province China

**Keywords:** coronary heart disease, meta‐analysis, mhealth, mobile health, randomized controlled trials, systematic review

## Abstract

The association between the efficacy of mobile health and the occurrence and development of coronary heart disease (CHD) is still unclear. Mobile health can alleviate the risk factors for CHD. PubMed, EMbase, Web of Science, The Cochrane Library, CNKI, WanFang, and VIP databases were searched from inception through May 28, 2020. Randomized controlled trials of the effect of mobile health in alleviating the risk factors of CHD's occurrence and development were included. Risks of bias were assessed by two independent reviewers by using the RevMan 5.3, GRADEpro, and RoB2.0 to generate findings. Meta‐analyses were performed to investigate the effects of mobile health on risk factors for CHD. Subgroup analyses were conducted. Sixteen randomized controlled trials, including 3898 patients with CHD, were included. Meta‐analysis results showed that mobile health can reduce BMI (mean difference [MD] = − 1.24, 95% CI = − 2.02 to − 0.45, p < .05), waist circumference (MD = − 4.40, 95% CI = − 4.72 to − 4.08, p < .00001), total cholesterol (TC) level (MD = − 0.43, 95% CI = − 0.64 to − 0.22, p < 0.00001), low‐density lipoprotein cholesterol (LDL‐C) level (MD = − 0.31, 95% CI = − 0.48 to − 0.15, p < .05), diastolic blood pressure (MD = − 2.01, 95% CI = − 3.40 to − 0.623, p < .05), and depression (MD = − 8.32, 95% CI = − 12.83 to − 3.81, p < .05) and increase high‐density lipoprotein cholesterol level (MD = 0.16, 95% CI = 0.01 to 0.32, p < .05) with statistically significant differences. The results of subgroup analyses indicated that the simple mobile health intervention has more remarkable advantages in reducing BMI, TC, LDL‐C, and systolic blood pressure than the complex mobile health intervention. Mobile health can alleviate the risk factors for CHD and has a certain effect on the prevention and recovery of CHD. Simple mobile health has a remarkable advantage. Limited by the quantity and quality of included studies, future research enrolling high‐quality studies should be taken to verify the above conclusions.

## 1 INTRODUCTION

Coronary heart disease (CHD) refers to coronary arterial stenosis or obstruction caused by progressive coronary atherosclerotic lesions and the resulting myocardial ischemia or necrosis.^1^, ^2^ CHD is the main component of global cardiovascular disease.^3^ Given its high prevalence and mortality rate, CHD has become a notable public health concern.^4^ Studies have shown^5^ that atherosclerosis is an inflammatory disease, and sickness (such as high blood pressure and abnormal lipoprotein content) are risk factors for the occurrence and promotion of inflammation. According to the 2020 American Heart Association report,^6^ patients with CHD are at a high risk of recurring coronary events. The main causes of recurrence are hypertension, hypercholesterolemia, dyslipidemia, and obesity or overweight.^7^ Current CHD prevention guidelines give high priority to the intensive control of CHD risk factors.^8^ At present, clinicians guide patients to self‐monitor risk factors through health education and follow‐up. However, these efforts have limited success due to the lack of family medical equipment, medical knowledge deficit, and weak self‐supervision and management. The mobile health (mhealth) can overcome these limitations and can remotely monitor and guide patients' home rehabilitation through smartphone applications, wearable devices, and text messages. The mhealth improves the patients' lifestyle and quality of life and assists them in meeting their individual needs.^9^ This study has used the AMSTAR 2.0^10^ for systematic review/meta‐analysis, reported data in accordance with the Preferred Reporting Items for Systematic Reviews and Meta‐Analyses (PRISMA) statement, analyzed the influence of mhealth on CHD risk factors, and provided evidence of higher methodological and reporting quality for the effect of mhealth on alleviating CHD risk factors.

## 2 METHODS

### 2.1 Search strategy

This study is a systematic review and meta‐analysis performed in accordance with the PRISMA guidelines.^11^ The search was conducted by two independent reviewers. Relevant randomized controlled trials (RCTs) were identified by searching the PubMed, EMbase, Web of Science, The Cochrane Library, CNKI, WanFang, and VIP databases until May 2020 by using the medical subject heading (MeSH) terms and all synonyms of “coronary disease” in combination with the MeSH term and all synonyms of “mHealth”. In addition, clinical trial registration websites (i.e., http://www.Clinical Trial.gov and http://www.chictr.org.cn) were searched, and the references of the included literature were manually searched to supplement access to relevant literature. The search terms and strategy are provided in Appendix 1 (Supplementary Material). References to all identified publications were entered into the reference management software (NoteExpress, version V3.0).

### 2.2 Selection criteria

Studies were included in accordance with the following eligibility criteria: (1) Designed as a RCT or a cluster RCT; (2) published in English or Chinese until May 2020; (3) patients, irrespective of sex or age, who were already clinically diagnosed with CHD, had undergone revascularization (coronary artery bypass grafting or percutaneous coronary intervention [PIC]), or with angina pectoris; (4) mhealth interventions, including mobile applications, text messages, or wearable sensors, to treat and follow up patients with CHD (the duration, frequency or type of medical intervention is not limited); and (5) presence of a control group, including but not limited to usual care, routine health education, and follow‐up. Other treatments were required to be consistent between experimental and control groups.

Studies that were repeated, with unavailable full text, missing original research data, and data that could not be extracted were excluded.

### 2.3 Data extraction and endpoints

Two researchers screened the literature independently and performed data extraction and cross‐checking. Disagreements were resolved through discussion or negotiation with a third reviewer. Titles and abstracts were read first to exclude evidently irrelevant literatures. The full texts of the remaining articles were reviewed for final inclusion. If necessary, the original research authors were contacted by email or phone to obtain undetermined but important information. The following data were extracted: (1) Basic information of the included research (e.g., title, author, journal, and year), (2) baseline characteristics and intervention measures, (3) primary outcome indicators (i.e., risk factors, including BMI, waist circumference, hips circumference, total cholesterol, low‐ [LDL‐C] and high‐density [HDL‐C] lipoprotein cholesterol, and systolic [SBP] and diastolic [DBP] blood pressures), and (4) secondary outcome indicators (i.e., emotional state, including anxiety and depression).

### 2.4 Data analysis and synthesis

The RevMan5.3 software was used for statistical analysis. Measurement data used the mean difference (MD) as the effect analysis statistics, and each effect size provided 95% CI. The heterogeneity among the results of the included studies was analyzed using the χ2 test (test level: *α* = 0.1), and the degree of heterogeneity was quantitatively judged in combination with *I* ^2^. The fixed‐effects model was used for analysis if no statistical heterogeneity among the results of each study was observed. If a statistical heterogeneity was observed among the results of each study, the source of the heterogeneity was further analyzed. After the exclusion of the influence of evident clinical heterogeneity, the random‐effects model was used for analysis. The level of meta‐analysis was set to *α* = 0.05. Evident clinical heterogeneity was treated using the subgroup analysis.

### 2.5 Study quality assessments

The included studies' risk of bias was evaluated independently and cross‐checked by two investigators. Assessments were performed using the GRADEpro and the RCT bias risk assessment tool ROB2.0, which was recommended by the Cochrane manual.^12^

## 3 RESULTS

### 3.1 Study selection

**FIGURE 1:**
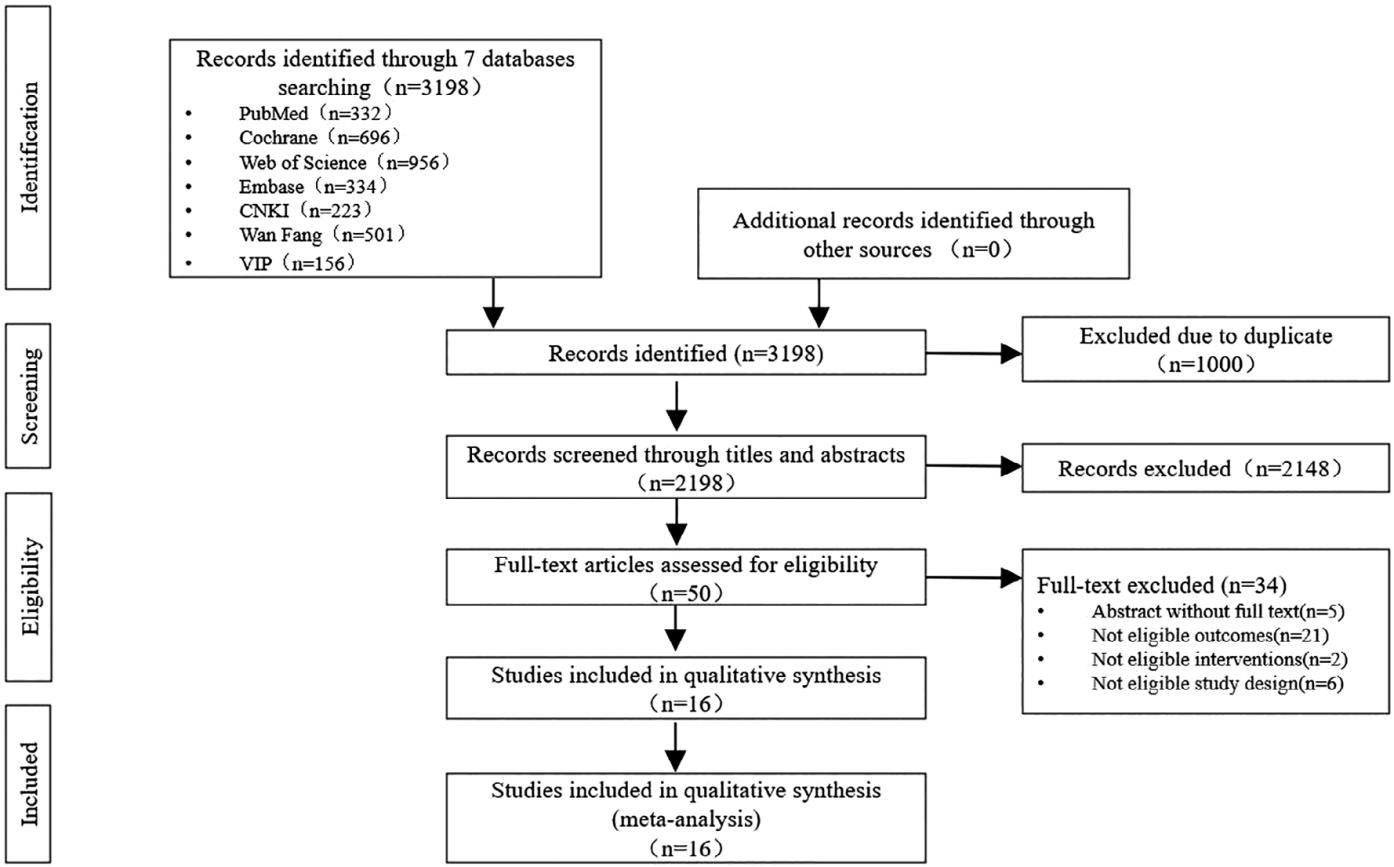
Preferred reporting items for systematic reviews and meta‐analyses (PRISMA) flow diagram

Figure 1 illustrates the PRISMA flow diagram in the systematic search process.

A total of 3198 articles were retrieved from seven databases, and 1000 articles were duplicates. By reading the title, abstract, and keywords, 2148 articles were screened for inappropriate intervention measures, population, and non‐RCTs. The remaining 50 full‐text articles were assessed independently against the eligibility criteria by two researchers. A total of 34 articles were excluded. Five studies were unable to obtain the full text due to language. The researcher tried to contact the author but got no response. The primary outcome indicators of 21 articles did not meet the inclusion criteria. Two articles had participants that did not match. The study types of six articles did not match. Finally, 16 RCTs, including 3898 patients with CHD, were included.^13^, ^14^, ^15^, ^16^, ^17^, ^18^, ^19^, ^20^, ^21^, ^22^, ^23^, ^24^, ^25^, ^26^, ^27^, ^28^

### 3.2 Description of included studies

Table 1 summarizes the characteristics of included studies. Sixteen trials, including 10 Chinese and 6 English articles, were published between 2011 and 2019. The majority of the subjects came from hospitals. All patients had CHD and undergone CHD interventional therapy and postoperative PIC. About 52.57% (2049/3898) of patients were male, and the average age ranged from 38.32 to 71.6 years. The PICO information is presented in Table 2.

**TABLE 1:** Characteristics of the included studies

Author, year	Country	Sample (I/C)	M/F (intervention group)	M/F (control group)	Age(I/C)	Population source	Duration (week)	Frequency
Fang, 2019^13^	China	33/34	12/21	21/13	60.24 ± 9.351/61.41 ± 10.169	Hospital	6	3 Times per week
Ge, 2019^14^	China	133/133	117/16	115/18	56.7 ± 10.0/56.5 ± 9.0	Hospital	48	—
Chow, 2015^15^	Australia	352/358	229/123	295/63	57.9 ± 9.1/57.3 ± 9.3	Hospital	24	4 Messages per week
Escobar, 2017^16^	Spain	14/14	14/0	14/0	56.50 ± 6.01/55.64 ± 11.35	Hospital	8	—
Santo, 2019^17^	Australia	107/56	93/14	50/6	58.4 ± 9.04/56.8 ± 8.64	Hospital	12	Daily
Blasco, 2012^18^	Spain	102/101	83/19	80/21	60.6 ± 11.5/61 ± 12.1	Hospital	48	Once a week
Nolan, 2011^19^	Canada	413/267	211/202	135/132	59.27 ± 0.43/58.61 ± 0.53	Community	24	6 Times per week
Avila, 2018^20^	Belgium	28/26	24/4	23/3	58.6 ± 13/61.7 ± 7.7	Hospital	12	Once a week
Zheng, 2019^21^	China	411/411	353/58	353/58	56.25 ± 9.3/56.56 ± 9.7	Hospital	24	6 Messages per week
Xu, 2016^22^	China	45/41	20/25	16/25	55. 1 ± 3. 3/58. 1 ± 1. 8	Hospital	48	—
Tian, 2019^23^	China	42/41	22/20	19/22	55.09 ± 5.14/54.86 ± 5.37	Hospital	12	Daily
Geng, 2015^25^	China	40/40	38/42		38.32 ± 5.21	Hospital	12	Twice a week
Zhang, 2018^26^	China	60/60	34/26	35/25	71.4 ± 2.6	Hospital	12	First month: 4 times; 2th‐3th month: twice a month
Zhang, 2017^27^	China	63/61	39/22	44/19	54.14 ± 8.66/52.84 ± 8.74	Hospital	12\24	First month: twice; 2th‐6th month: once a month
Dorje, 2019^28^	China	156/156	128/28	126/30	59.1 ± 9.4/61.9 ± 8.7	Hospital	24	1th‐2th month: 4 times per week; 3th‐6th month: twice a week
Bai, 2019^24^	China	50/50	31/19	29/21	70.8 ± 4.0/71.6 ± 3.8	Hospital	28	Daily

**TABLE 2:** PICO of the included studies

Author, year	P	I	C	O
Fang, 2019^13^	post‐PCI patients	CHD educational booklet; complete outdoor walking or jogging with real‐time physiological monitoring; home visits; telephone call	Usual care (paper‐based CHD educational booklets)	⑦⑧
Ge, 2019^14^	post‐PCI patients	Exercise prescription, remote heart rate supervision and rehabilitation guidance from rehabilitation physicians, technicians and nurses with aid of smart phones	Usual care (health education; drug therapy)	⑦⑧④⑤
Chow, 2015^15^	CHD patients	Text messages	Usual care	①②③④⑤⑦
Escobar, 2017^16^	CHD patients	In‐home exercises monitored with a remote electrocardiographic monitoring device (NUUBO); traditional cardiac rehabilitation	Traditional cardiac rehabilitation	①④⑤⑥⑦⑧
Santo, 2019^17^	CHD patients	Medication reminder apps; usual care	Usual care (cardiovascular medications prescription, lifestyle advice and referral to cardiac rehabilitation)	④⑤⑥⑦
Blasco, 2012^18^	acute coronary syndrome patients	Individualized short message service text messages with recommendations	Lifestyle counseling; usual care treatment	①⑤⑦⑧
Nolan, 2011^19^	at high risk or with CHD	Teleconferenced sessions; active control	Active control (risk factor feedback, brief advice, handouts)	⑦⑧
Avila, 2018^20^	CAD Patients	Home‐based exercise intervention with telemonitoring guidance consisting of emails or phone calls	Usual care (advice to remain physically active)	①②③④⑤⑥
Zheng, 2019^21^	CHD patients	Text messages: general disease knowledge of CHD, BP control, medication adherence, physical activity, healthy diet, and smoking cessation	A personalized welcoming message, a birthday greeting, and a follow‐up reminder	①⑤⑦⑧
Xu, 2016^22^	CHD patients	Health education via WeChat (medication reminder, individual exercise program, communication, feedback)	Usual care (oral explanation; distribution of relevant materials)	①④
Tian, 2019^23^	CHD patients	Continued intervention based on WeChat (CHD knowledge, health guidance, telephone follow‐up)	Usual discharge guidance, regular telephone follow‐up	④⑦⑧
Geng, 2015^25^	CHD patients	Follow WeChat official account (Send nursing knowledge about CHD, in the form of text, pictures, videos); Join WeChat group (Communication, consultation, psychological counseling, and guiding patients to perform rehabilitation exercises)	Usual discharge health education(Medication guidance, diet guidance, pain and life care, functional exercise)	⑨⑩
Zhang, 2018^26^	Elderly CHD patients	Follow WeChat official account (medication reminder; CHD knowledge); Join WeChat group (Communication, consultation, patient experience sharing)	Telephone interview	⑨⑩
Zhang, 2017^27^	post‐PCI patients	Follow‐up management with smart phone app (Follow‐up, personalized health education, communication and feedback)	Usual care	④⑤⑥
Dorje, 2019^28^	post‐PCI patients	SMART‐CR/SP system (smartphone‐based home cardiac rehabilitation and secondary prevention programme delivered via the WeChat platform)	Standard outpatient cardiology follow‐up	①④⑤⑥⑦
Bai, 2019^24^	CHD patients	Information management beyond the hospital by Android‐based cardiac rehabilitation	Usual medical treatment	①④⑤⑥

*Note*: ①BMI②Waist circumference③Hip circumference④Total Cholesterol⑤LDL‐C⑥HDL‐C⑦Systolic blood pressure⑧Diastolic blood pressure ⑨Anxiety ⑩Depression.

Abbreviation: CHD, coronary heart disease.

### 3.3 Quality appraisal

This article uses the ROB2.0 to evaluate the literature. The risk of bias is shown in Supplementary Figures 1 and 2. Three trials^15^, ^17^, ^19^ (18.75%) were rated to have low risk of bias for the randomization process. Nine trials^14^, ^15^, ^21^, ^22^, ^23^, ^24^, ^25^, ^26^, ^28^ (56.25%) were rated to have low risk of bias for deviations from intended interventions. All trials were rated to have low risk of bias for missing outcome data, measurement of the outcome, and selection of the reported result. One trial^15^ (6.25%) was rated to have low risk of bias for overall bias. In general, all studies have low or medium risk of bias due to insufficient reporting on the randomization or the allocation process. In most studies, blinding was not implemented and applicable for subjects, but the lack of blinding was unlikely to have influenced the assessment of primary outcome indicators.

**FIGURE 2:**
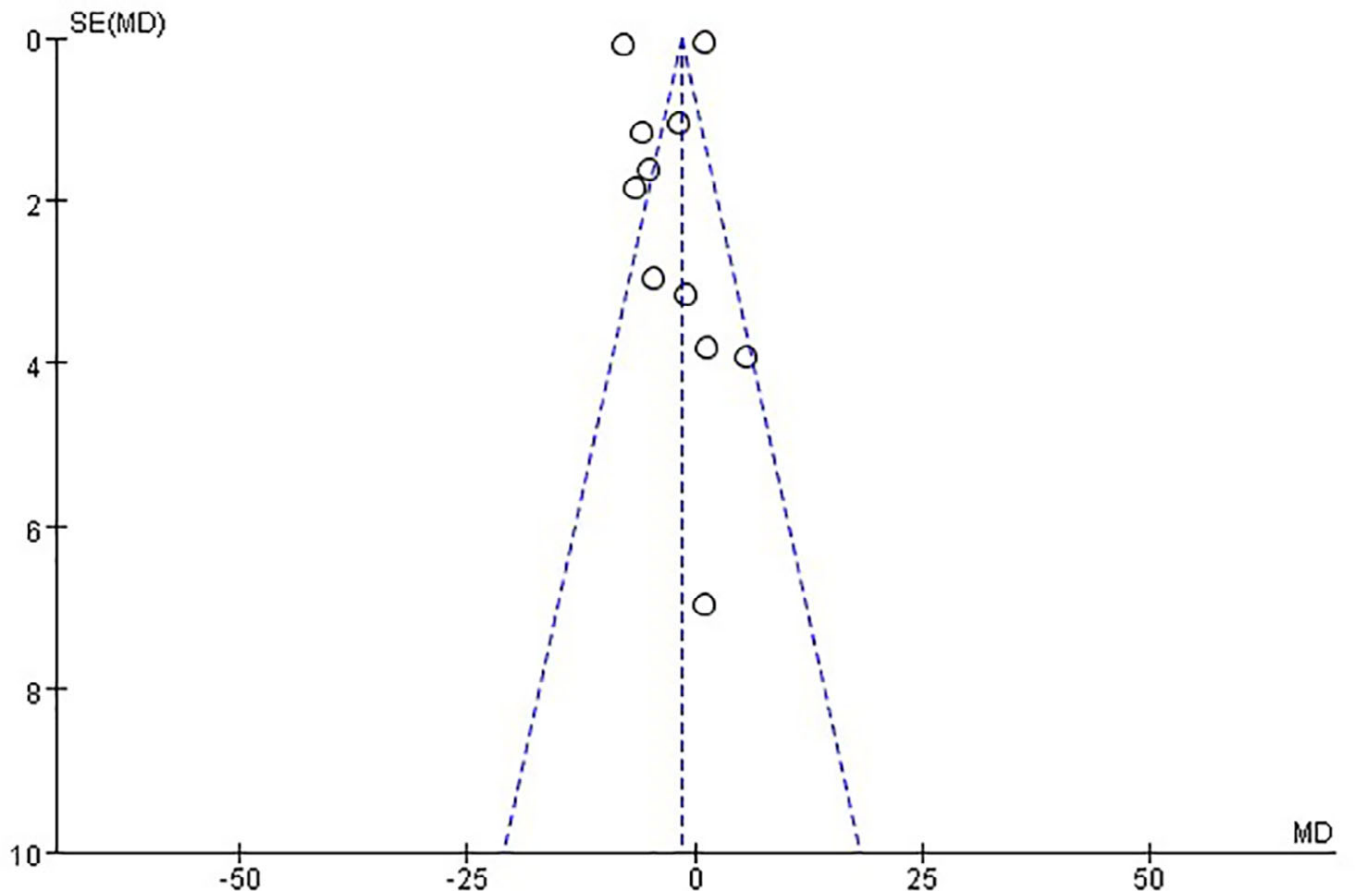
Funnel plots: Publication bias of included articles

Funnel plots were applied to evaluate the publication bias of the study, which included more than 10 articles. In this study, the SBP was incorporated into the literature to make the funnel chart. Results showed partial asymmetry, suggesting the possibility of publication bias (Figure 2).

### 3.4 Meta‐analysis for outcome measures

In the included studies, a meta‐analysis of three risk factors, namely, obesity, cholesterol, and blood pressure and emotions, was performed. The data evaluated in each field were summarized as follows.

### 3.5 Obesity

#### 3.5.1 BMI

Seven RCTs,^15^, ^16^, ^20^, ^21^, ^22^, ^24^, ^28^ consisting of 1699 patients with CHD, were included. The analysis results of the random‐effects model showed that the body mass index (BMI) of the mhealth group was lower than that of the control group (MD = − 1.24, 95% CI = − 2.02 to − 0.45, p = .002; Supplementary Figure 3).

#### 3.5.2 Waist circumference

Two RCTs^15^, ^20^ with 764 patients were included. The results of the fixed‐effects model analysis showed that the waist circumference of the mhealth group was lower than that of the control group (MD = − 4.40, 95% CI = − 4.72 to − 4.08, p < .00001; Supplementary Figure 4).

#### 3.5.3 Hip circumference

Two RCTs^15^, ^20^ with 764 patients were included. The random‐effects model analysis showed that the hip circumference of the mhealth group was lower than that of the control group (MD = − 3.18, 95% CI = − 6.66 to 0.31, p = .07; Supplementary Figure 5).

### 3.6 Cholesterol

#### 3.6.1 Total cholesterol

Eight RCTs^15^, ^16^, ^17^, ^22^, ^24^, ^27^, ^28^ with 1262 patients were included. The results of the random‐effects model analysis showed that the total cholesterol level in the mhealth group was lower than that in the control group (MD = − 0.43, 95% CI = − 0.64 to − 0.22, p < .0001; Supplementary Figure 6).

#### 3.6.2 Low‐density lipoprotein cholesterol

Seven RCTs with 1915 patients ^15^, ^16^, ^17^, ^21^, ^24^, ^27^, ^28^ were included. The results of the random‐effects model analysis showed that the LDL‐C level of the mhealth group was lower than that of the control group (MD = − 0.31, 95% CI = − 0.48 to − 0.15, p = .0001; Supplementary Figure 7).

#### 3.6.3 High‐density lipoprotein cholesterol

Five RCTs^15^, ^16^, ^24^, ^27^, ^28^ with 861 patients enrolled were included. The results of the random‐effects model analysis showed that the HDL‐C level of the mhealth group was higher than that of the control group (MD = 0.16, 95% CI = 0.01 to 0.32, p = .03; Supplementary Figure 8).

### 3.7 Blood Pressure

#### 3.7.1 Systolic blood pressure

Eleven RCTs^13^, ^14^, ^15^, ^16^, ^17^, ^18^, ^19^, ^20^, ^21^, ^23^, ^28^ with 3031 patients were included. The random‐effects model analysis showed that the SBP of the mhealth group was lower than that of the control group (MD = − 2.61, 95% CI = − 6.60 to 1.38, p = .20; Supplementary Figure 9).

#### 3.7.2 Diastolic blood pressure

Nine RCTs^13^, ^14^, ^15^, ^16^, ^17^, ^18^, ^19^, ^20^, ^23^ with 2209 patients were included. The random‐effects model analysis showed that the DBP of the mhealth group was lower than that of the control group (MD = − 2.01, 95% CI = − 3.40 to − 0.62, p = .005; Supplementary Figure 10).

### 3.8 Emotions

#### 3.8.1 Anxiety

Two RCTs^25^, ^26^ with 200 patients were included. The random‐effects model analysis showed that the anxiety of the mhealth group was lower than that of the control group (MD = 17.21, 95% CI = − 30.89 to 65.31, p = .48; Supplementary Figure 11).

#### 3.8.2 Depression

Two RCTs^25^, ^26^ with 200 patients were included. The random‐effects model analysis showed that the depression of the mhealth group was lower than that of the control group (MD = − 8.32, 95%CI = − 12.83 to − 3.81, p = .0003; Supplementary Figure 12).

### 3.9 Subgroup analysis

Meta‐analysis results showed that many outcomes had *I* ^2^>50%, suggesting a large heterogeneity among studies. The subgroup analysis of 16 studies was carried out in accordance with different mhealth intervention methods to clarify the source of heterogeneity. The interventions received by the simple mhealth group, which consisted of eight articles,^15^, ^18^, ^20^, ^22^, ^23^, ^24^, ^25^, ^26^ included telephone calls, messages, and WeChat messages. The interventions received by the complex mhealth group, which consisted of eight articles,^13^, ^14^, ^16^, ^17^, ^19^, ^20^, ^27^, ^28^ included self‐developed applications, wearable devices, and video conferencing.

#### 3.9.1 BMI

The heterogeneity test after merging still showed a large heterogeneity. Thus, the random‐effects model was used for analysis. Four studies^15^, ^21^, ^22^, ^24^ were included in the simple mhealth group and indicated that the simple mhealth intervention was significantly better than the control in reducing BMI (MD = − 1.71, 95% CI = − 2.66 to − 0.77, p = .0004). Three studies^16^, ^20^, ^28^ were included in the complex mhealth group and indicated that the complex mhealth intervention could not significantly reduce BMI (MD = − 0.21, 95%CI = − 1.39 to 0.97, p = .72). Results are shown in Supplementary Figure 13.

#### 3.9.2 Total cholesterol

Four studies^15^, ^22^, ^23^, ^24^ were included in the simple mhealth group and showed that the simple mhealth intervention was significantly better than the control in reducing the total cholesterol level (MD = − 0.65, 95% CI = − 0.88 to − 0.42), p < .00001). Four studies^16^, ^17^, ^27^, ^28^ were included in the complex mhealth group and showed that the complex mhealth group could not significantly reduce the total cholesterol level (MD = − 0.11, 95% CI = − 0.41 to 0.19, p = .48; Supplementary Figure 14).

#### 3.9.3 Low‐density lipoprotein cholesterol

Three studies^15^, ^21^, ^24^ were included in the simple mhealth group and showed that the simple mhealth intervention was significantly better than the control in reducing the LDL‐C level (MD = − 0.40, 95% CI = − 0.63 to − 0.18, p = .0004). Four studies^16^, ^17^, ^27^, ^28^ were included in the complex mhealth group and indicated that the complex mhealth intervention could not significantly reduce the LDL‐C level (MD = − 0.24, 95%CI = − 0.57 to 0.08, p = .14; Supplementary Figure 15).

#### 3.9.4 Systolic blood pressure

Four studies^15^, ^18^, ^21^, ^23^ were included in the simple mhealth group and indicated that the simple mhealth intervention was significantly better than the control in reducing SBP (MD = − 5.18, 95% CI = − 8.64 to − 1.72, p = .003). Seven studies^13^, ^14^, ^16^, ^17^, ^19^, ^20^, ^28^ were included in the complex mhealth group and indicated that the complex mhealth group could not significantly reduce SBP (MD = − 1.27, 95% CI = − 4.72 to 2.18, p = .47; Supplementary Figure 16).

#### 3.9.5 Diastolic blood pressure

Three studies^15^, ^18^, ^23^ were included in the simple mhealth group and showed that the simple mhealth intervention was significantly better than the control in reducing DBP (MD = − 3.30, 95% CI = − 5.23 to − 1.38, p = .0008). Six studies^13^, ^14^, ^16^, ^17^, ^19^, ^20^ were included in the complex mhealth group and showed that the complex mhealth intervention could significantly reduce DBP (MD = − 0.78, 95% CI = − 0.88 to − 0.69, p < .00001; Supplementary Figure 17).

### 3.10 GRADEpro evidence assessment

The GRADEpro was used to evaluate the evidence. In terms of the limitations of the study, the blinding and the allocation concealment reported in some included studies were insufficient, had little effect on experimental results, and were not downgraded. In terms of inconsistency, most indicators were statistically heterogeneous and downgraded. In terms of indirectness, although the included intervention measures were not completely consistent, no significant difference was observed between PICO and the research aim. Thus, it has not be downgraded. In terms of inaccuracies, some of the outcome sample sizes did not meet the optimal sample size. Some of the outcome effect sizes crossed the invalid line and should be downgraded. In terms of publication bias, the included literature did not involve commercial interests. The literature was searched comprehensively. No clear evidence currently showed a risk of bias, and it was not downgraded. Results are summarized in Table 3.

**TABLE 3:** Evidence assessment of outcomes

Outcomes (studies)	Anticipated absolute effects (95% CI)	Patients (T/C)	Quality of evidence	Rated down reasons
BMI (7)	MD = ‐1.24(−2.02 to −0.45)	1055/1056	⊕⊕⊕◯ MODERATE	Inconsistency
Waist‐circumstance (2)	MD = ‐4.40(−4.72 to −4.08)	380/384	⊕⊕⊕⊕HIGH	
Hip‐circumstance (2)	MD = ‐3.18(−6.66 to 0.31)	380/384	⊕⊕◯◯LOW	Inconsistency; Imprecision
TC (8)	MD = ‐0.43(−0.64 to −0.22)	822/772	⊕⊕⊕◯ MODERATE	Inconsistency
LDL‐c (7)	MD = ‐0.31(−0.48 to −0.15)	1146/1101	⊕⊕⊕◯ MODERATE	Inconsistency
HDL‐c (5)	MD = 0.16(0.01 to 0.32)	634/639	⊕⊕⊕◯ MODERATE	Inconsistency
SBP (11)	MD = ‐2.61(−6.6 to 1.38)	1769/1574	⊕⊕◯◯LOW	Inconsistency; Imprecision
DBP (9)	MD = ‐2.01(−3.4 to 0.62)	1202/1007	⊕⊕◯◯LOW	Risk of bias; Inconsistency
Anxiety (2)	MD = ‐17.21(−30.89 to 65.31)	100/100	⊕◯◯◯VERY LOW	Risk of bias; Inconsistency; Imprecision
Depression (2)	MD = ‐2.01(−3.4 to 0.64)	100/100	⊕◯◯◯VERY LOW	Risk of bias; Inconsistency; Imprecision

Abbreviations: HDL‐C, high‐density lipoprotein cholesterol; LDL‐C, low‐density lipoprotein cholesterol; SBP, systolic blood pressures.

## 4 DISCUSSION

Through the GRADE classification, this study has obtained evidence of different quality levels and demonstrated that the mhealth has a certain effect on alleviating the risk factors that cause the occurrence and development of CHD. In terms of obesity, high‐quality evidence indicates that the mhealth can narrow the waist circumference of patients with CHD and play a remarkable role in controlling obesity. Moderate‐quality evidence indicates that the mhealth reduces the BMI of patients with CHD. Low‐quality evidence suggests that the mhealth has no significant effect on the hip circumference. In terms of blood pressure, low‐quality evidence suggests that the mhealth can decrease the DBP but has no significant effect on the SBP. In terms of serum lipid, moderate‐quality evidence shows that the mhealth significantly reduces total cholesterol and LDL‐C levels and increases the HDL‐C level. In terms of emotions, low‐quality evidence shows that the mhealth can relieve depression in patients with CHD but has no effect on anxiety.

Obesity is an independent risk factor for the occurrence and development of cardiovascular diseases.^29^ Compared with nonobese people, obese people have faster development of coronary atherosclerosis, and the incidence of cardiovascular disease differs due to fat distribution.^30^ A previous study^31^ shows that obesity‐related indicators from the intervention group are significantly reduced after ≥24 weeks. In this study, the short‐term mhealth intervention can reduce the patient's BMI and waist circumference but does not significantly improve the hip circumference, which may be related to the difference in the patient's fat distribution. A reasonable intake of diet can help patients control their body. Studies have confirmed that low‐salt, low‐fat, and healthy eating habits can significantly reduce the incidence of CHD by 20%–33%, delay the progression of atherosclerosis, reduce cardiovascular and metabolic risk factors, and improve the quality of life.^32^, ^33^

Dyslipidemia is one of the prominent risk factors for CHD.^34^ Increased LDL‐C level and decreased HDL‐C level may induce CHD.^35^, ^36^ The increase in LDL‐C level causes arterial intimal injury, which causes fibrocyte‐producing proliferative reactions and eventually develops into atherosclerosis.^37^ The reduction in HDL‐C level affects peripheral tissues, thereby affecting the antiatherosclerosis effect.^38^ In this study, after the intervention of the mhealth, total cholesterol and LDL‐C levels decrease significantly, and HDL‐C level increases. These results are similar to the results of Dale,^39^ indicating that the mhealth treatment is important to alleviate the occurrence and development of CHD. However, study^40^ has shown that, compared with changes in cholesterol, 10 year ASCVD risk can better reflect the close relationship between blood lipids and the incidence of coronary heart disease. There is no research related to 10 year ASCVD risk in the literature included in this study, and it can be combined with mhealth research in the future.

A large global prospective study shows that blood pressure levels are positively correlated with the incidence of CHD.^41^ The long‐term abnormal blood pressure can cause damage to the vascular endothelial function and induce atherosclerosis.^42^ Research^43^ points out that after 18 weeks of WeChat intervention, the DBP of patients is significantly decreased, whereas the SBP does not take effect until 24 weeks. In this study, the mhealth significantly improves the DBP, but the effect on the SBP is not evident. This finding may be related to the relatively short intervention time of included studies. Studies indicate that^44^, ^45^ strong emotions especially negative emotions, like anger, hostility, depression, and stress, can precipitate CHD. Therefore, the mental state of patients during treatment and recovery should be given attention. The anxiety level of the experimental group in this study is not significantly different from that of the control group. This finding is inconsistent from the conclusions of some scholars^46^ and may be related to the small number of the included literature, small sample size, and large heterogeneity. After the mhealth intervention, the depression level of the patient decreases significantly. This finding is in accordance with the results of Grossman^47^ and may be linked to the ability of the mhealth to pay close attention to patients remotely and answer their questions at any time. The wearable device can report the patient's health data in real time. Thus, the patient can accurately perceive their physical condition, thereby reducing concerns about the disease.

In addition, smoking^48^ and blood sugar levels are important factors leading to the occurrence and development of CHD. A previous study^49^ has confirmed a direct linear relationship between blood glucose levels and the mortality of hospitalized patients with myocardial congestion and an effect on long‐term prognosis. These two points are not included in this study, which may be because most researchers are not focused on the combination of mhealth, smoking, and blood sugar. Although not covered in this study, the effects of smoking and blood sugar on CHD should be given attention. Future studies should incorporate these two aspects to consider the role of the mhealth.

Included studies are divided into two subgroups, that is, simple and complex mhealth intervention groups, to clarify the source of heterogeneity for in‐depth analysis. The simple mhealth intervention group is sent text or WeChat messages for health education and rehabilitation guidance. Simple methods, such as text, voice, and pictures, are used to convey information. The complex mhealth intervention group is contacted through applications, wearable devices, medical platforms, and real‐time video communication provided by researchers for interaction, feedback, and diversified home rehabilitation management. The results of the subgroup analysis indicate that the simple mhealth group shows remarkable advantages in reducing BMI, total cholesterol level, LDL‐C level, SBP, and DBP. This finding may be related to the CHD predilection. CHD occurs frequently in middle‐aged and elderly people.^50^ Older people have fairly low levels of education with reduced cognitive ability and memory, lack of effective learning methods for mobile devices and Internet applications, short exposure to the Internet, and weak cognitive ability. A previous study suggests that older adults are from a different technological generation and find it difficult to use technological devices nowadays.^51^ Thus, older people have difficulty in accepting and adapting to cumbersome informatization interventions. The complicated and small font size of the interface design of some complex mhealth interventions or the difficult operation process of the wearable device may reduce the enthusiasm of older people. Studies^51^, ^52^ show that older people use the Internet by focusing on communication and information acquisition. This finding is consistent with the intervention methods of the simple mhealth group, making older adults easily accept health information delivered by SMS, WeChat, and email. Thus, attention should be paid to the particularity of the receptivity, memory, and audition ability of the elderly. The improvement in mobile medical software and hardware, such as enlargement of interface fonts and reduction of wearable programs, should be strengthened to make the comprehensive home rehabilitation management accessible and understandable, facilitate the understanding and operation of older people, increase their enthusiasm for participation, and improve the display complexity of the mhealth's unique role in preventing and treating CHD.

### 4.1 Limitation

Some shortcomings remain in this study. First, the great heterogeneity in the type of intervention ranging from SMS to mobile application and wearables can be a great contributor to the variation of outcomes among studies. Furthermore, the remission of CHD risk factors is closely related to the intervention duration. However, the compliance with each intervention may be a confounder in long‐term studies. Considering that the intervention duration of the included studies is relatively short, the subgroup analysis cannot be conducted on the basis of the duration factor to explore long‐term mhealth intervention. At the same time, CHD‐related emotional indicators are rarely included in the literature, whereas the quality of life, anxiety, and depression are also important factors that influence the occurrence and the recurrence of CHD. Thus, subsequent data collection for this aspect should be strengthened. The RCTs included in this study generally lack standard allocation concealment and blinding because they are not applicable. Although it was not downgraded in this study, further research is needed to strengthen the methods of randomization, allocation, and blinding to improve the quality of evidence.

## 5 CONCLUSION

Research conclusions point out that the mhealth can alleviate the occurrence and development of risk factors (such as obesity, cholesterol, and DBP) and depression in patients with CHD. This study further clarifies the effect of different types of mhealth treatments on CHD risk factors. The results of the subgroup analysis show that the simple mhealth group is more conducive to controlling risk factors than the complex mhealth group. For elderly potential patients or patients with CHD, the application of simple remote mhealth is extensive in the future. However, given the limitations of the studies reviewed, the effects of mhealth on risk factors of CHD remain inconclusive. Studies with long‐term intervention and rigorous study design are needed to provide evidence.

## Supporting information


**Supplementary Figure 1** Risk of bias graph.

**Supplementary Figure 2** Risk of bias summary.

**Supplementary Figure 3** Forest plot: effectiveness of m‐health interventions on BMI.

**Supplementary Figure 4** Forest plot: effectiveness of m‐health interventions on waist circumference.

**Supplementary Figure 5** Forest plot: effectiveness of m‐health interventions on hip circumference.

**Supplementary Figure 6** Forest plot: effectiveness of m‐health interventions on total cholesterol.

**Supplementary Figure 7** Forest plot: effectiveness of m‐health interventions on LDL‐c.

**Supplementary Figure 8** Forest plot: effectiveness of m‐health interventions on HDL‐c.

**Supplementary Figure 9** Forest plot: effectiveness of m‐health interventions on SBP.

**Supplementary Figure 10** Forest plot: effectiveness of m‐health interventions on DBP.

**Supplementary Figure 11** Forest plot: effectiveness of m‐health interventions on anxiety.

**Supplementary Figure 12** Forest plot: effectiveness of m‐health interventions on depression.

**Supplementary Figure 13** Forest plot: effectiveness of different types of m‐health interventions on BMI.

**Supplementary Figure 14** Forest plot: effectiveness of different types of m‐health interventions on total cholesterol.

**Supplementary Figure 15** Forest plot: effectiveness of different types of m‐health interventions on LDL‐c.

**Supplementary Figure 16** Forest plot: effectiveness of different types of m‐health interventions on SBP.

**Supplementary Figure 17** Forest plot: effectiveness of different types of m‐health interventions on DBP.

**Appendix S1**: Search Strategies.
