# Supplementary figures and images for: The efficacy of mobile health in alleviating risk factors related to the occurrence and development of coronary heart disease: A systematic review and meta‐analysis
Source: Clin Cardiol. 2021 Mar 16;44(5):609–19. doi: 10.1002/clc.23596 (PMC8119799; doi:10.1002/clc.23596)

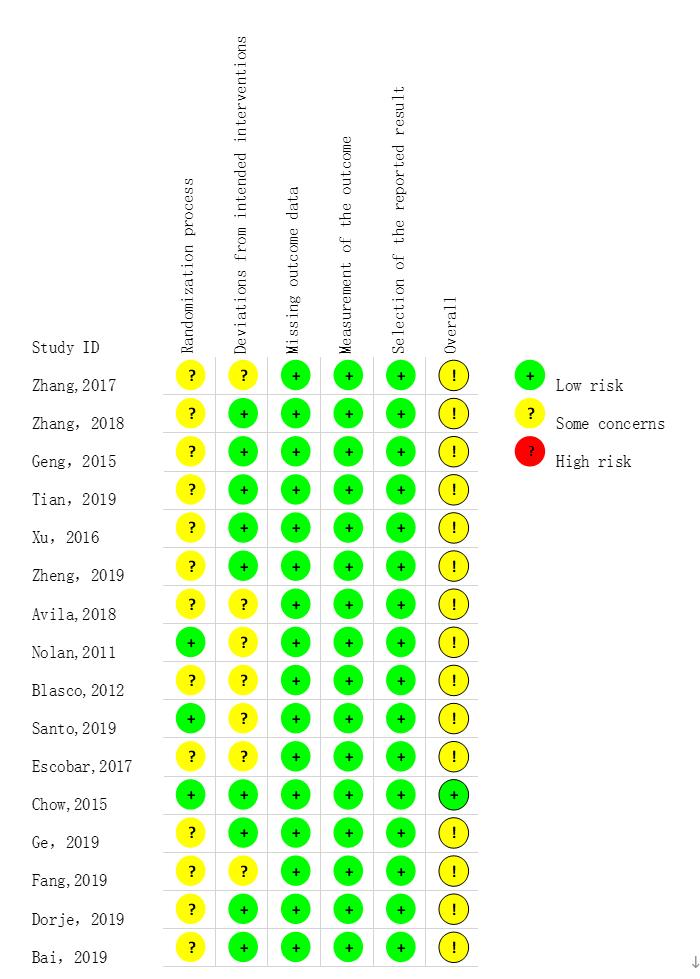

Supplement: Supplementary file 1 — Supplementary Figure 1 Risk of bias graph. [file CLC-44-609-s015.jpg]

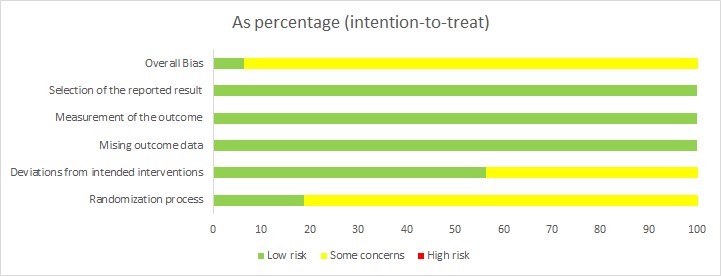

Supplement: Supplementary file 2 — Supplementary Figure 2 Risk of bias summary. [file CLC-44-609-s018.jpg]

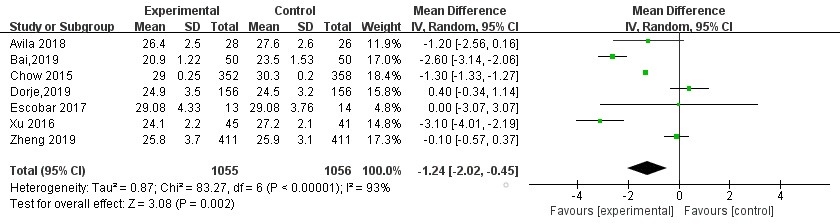

Supplement: Supplementary file 3 — Supplementary Figure 3 Forest plot: effectiveness of m‐health interventions on BMI. [file CLC-44-609-s007.jpg]

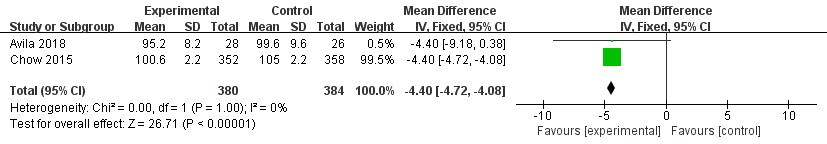

Supplement: Supplementary file 4 — Supplementary Figure 4 Forest plot: effectiveness of m‐health interventions on waist circumference. [file CLC-44-609-s003.jpg]

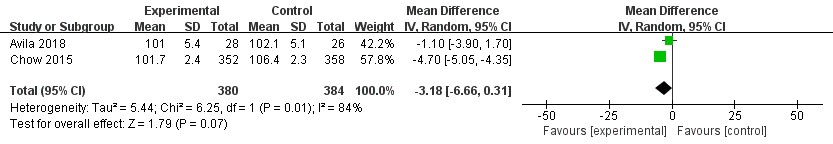

Supplement: Supplementary file 5 — Supplementary Figure 5 Forest plot: effectiveness of m‐health interventions on hip circumference. [file CLC-44-609-s005.jpg]

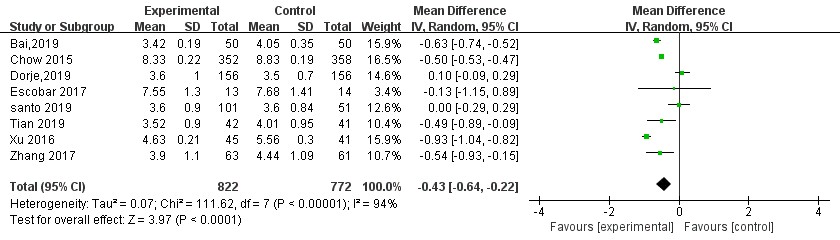

Supplement: Supplementary file 6 — Supplementary Figure 6 Forest plot: effectiveness of m‐health interventions on total cholesterol. [file CLC-44-609-s011.jpg]

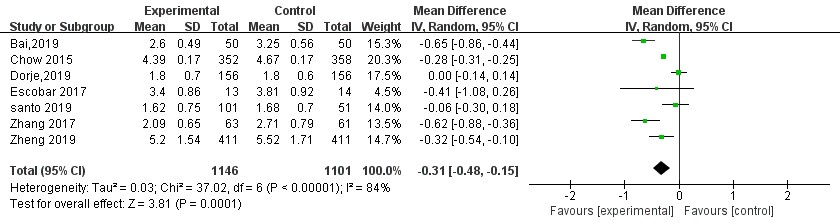

Supplement: Supplementary file 7 — Supplementary Figure 7 Forest plot: effectiveness of m‐health interventions on LDL‐c. [file CLC-44-609-s012.jpg]

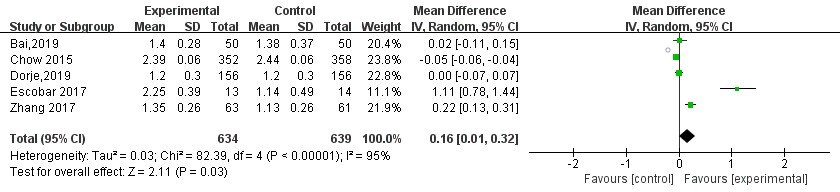

Supplement: Supplementary file 8 — Supplementary Figure 8 Forest plot: effectiveness of m‐health interventions on HDL‐c. [file CLC-44-609-s010.jpg]

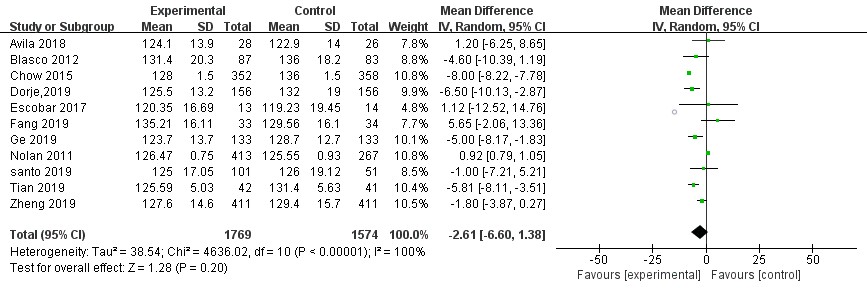

Supplement: Supplementary file 9 — Supplementary Figure 9 Forest plot: effectiveness of m‐health interventions on SBP. [file CLC-44-609-s016.jpg]

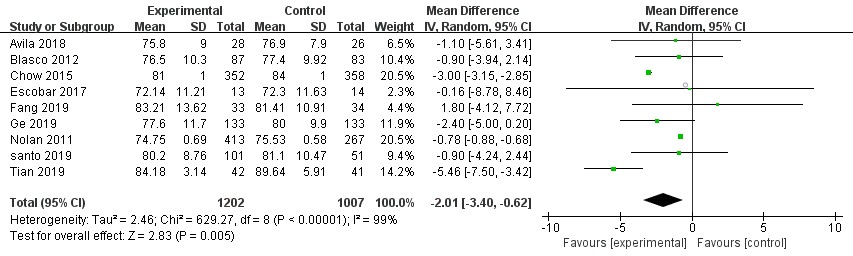

Supplement: Supplementary file 10 — Supplementary Figure 10 Forest plot: effectiveness of m‐health interventions on DBP. [file CLC-44-609-s009.jpg]

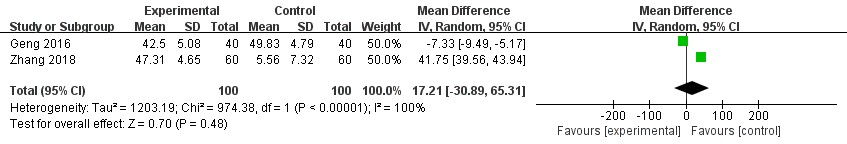

Supplement: Supplementary file 11 — Supplementary Figure 11 Forest plot: effectiveness of m‐health interventions on anxiety. [file CLC-44-609-s017.jpg]

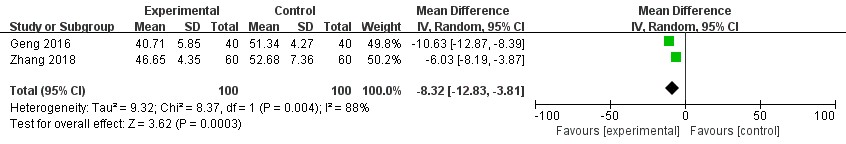

Supplement: Supplementary file 12 — Supplementary Figure 12 Forest plot: effectiveness of m‐health interventions on depression. [file CLC-44-609-s013.jpg]

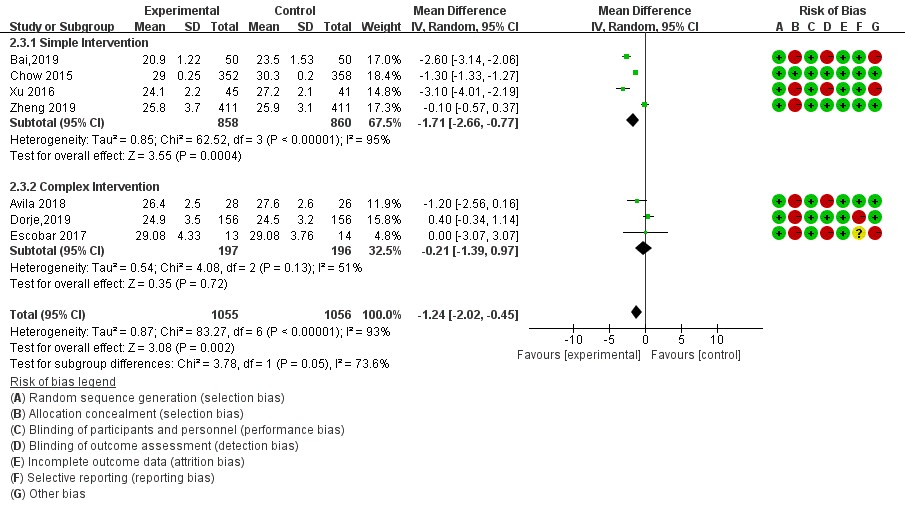

Supplement: Supplementary file 13 — Supplementary Figure 13 Forest plot: effectiveness of different types of m‐health interventions on BMI. [file CLC-44-609-s006.jpg]

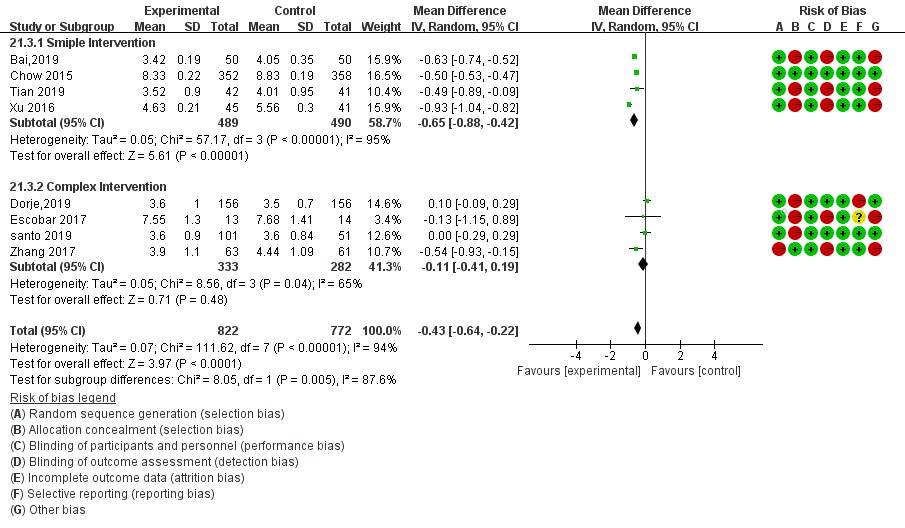

Supplement: Supplementary file 14 — Supplementary Figure 14 Forest plot: effectiveness of different types of m‐health interventions on total cholesterol. [file CLC-44-609-s004.jpg]

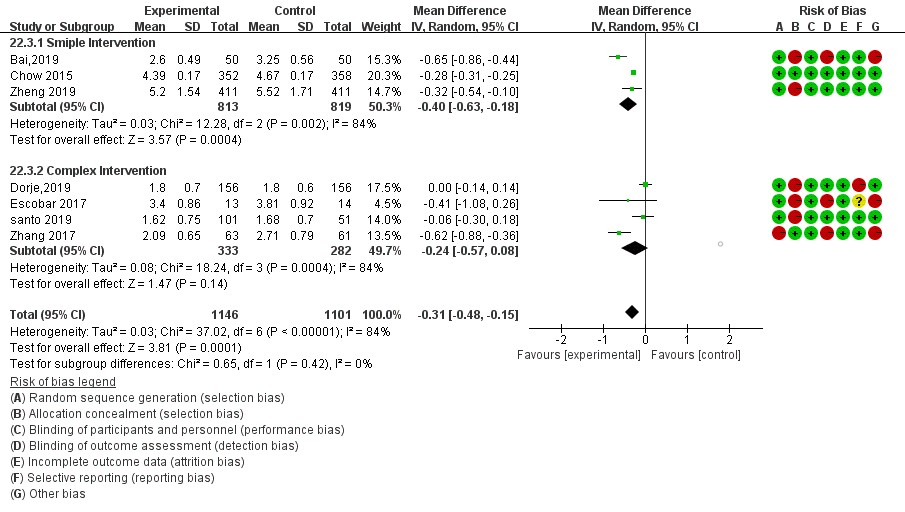

Supplement: Supplementary file 15 — Supplementary Figure 15 Forest plot: effectiveness of different types of m‐health interventions on LDL‐c. [file CLC-44-609-s002.jpg]

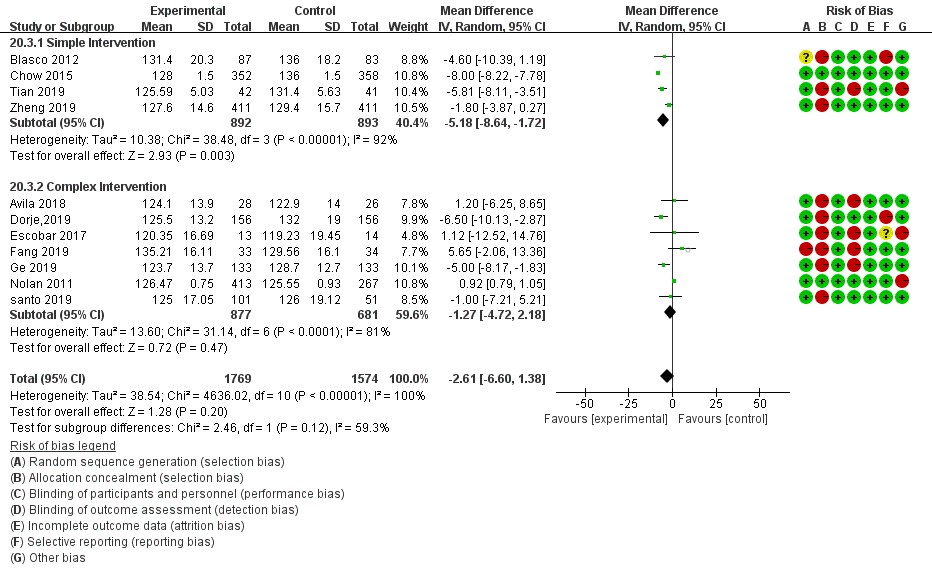

Supplement: Supplementary file 16 — Supplementary Figure 16 Forest plot: effectiveness of different types of m‐health interventions on SBP. [file CLC-44-609-s014.jpg]

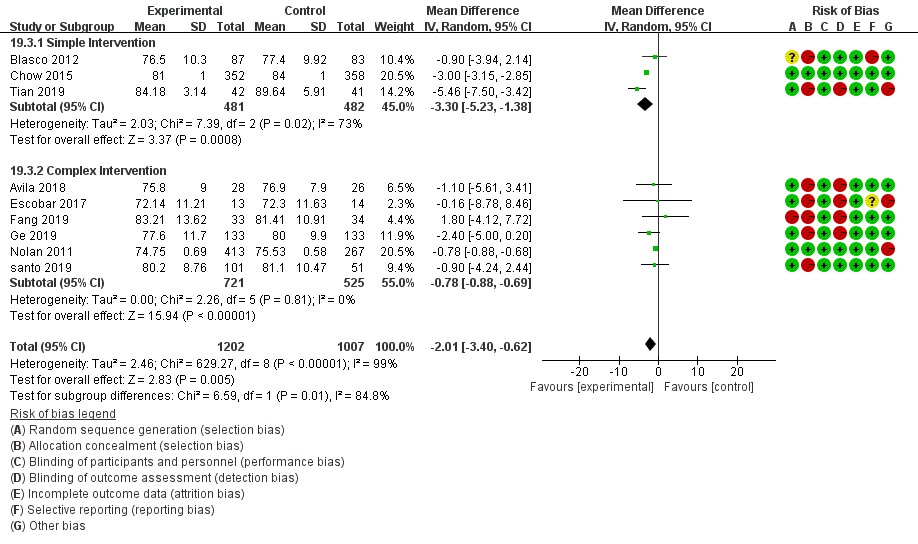

Supplement: Supplementary file 17 — Supplementary Figure 17 Forest plot: effectiveness of different types of m‐health interventions on DBP. [file CLC-44-609-s008.jpg]
