# Supplementary material for: The efficacy of mobile health in alleviating risk factors related to the occurrence and development of coronary heart disease: A systematic review and meta‐analysis
Source: Clin Cardiol. 2021 Mar 16;44(5):609–19. doi: 10.1002/clc.23596 (PMC8119799; doi:10.1002/clc.23596)
Supplement: Supplementary file 18 — Appendix S1: Search Strategies. [file CLC-44-609-s001.docx]

Appendix: Search Strategies

PubMed

| No. | Search Details | No. of Articles |
| --- | --- | --- |
| 1 | Telemedicine [Mesh] OR mobile health units [Mesh] | 33748 |
| 2 | mobile health [Title/Abstract] OR mHealth[Title/Abstract] OR Telehealth[Title/Abstract] OR eHealth[Title/Abstract] OR mobile applications [Title/Abstract] OR telemedicine [Title/Abstract] OR message [Title/Abstract] OR WeChat [Title/Abstract] OR application[Title/Abstract] OR mobile phone | 17279 |
| 3 | #1 OR #2 | 48998 |
| 4 | coronary disease [Mesh] | 218741 |
| 5 | coronary heart disease [Title/Abstract] OR coronary[Title/Abstract] OR CHD[Title/Abstract] OR Coronary Diseases [Title/Abstract] | 413352 |
| 6 | #4 OR #5 | 473927 |
| 7 | risk factors [Mesh] OR Treatment outcome[Mesh] | 1804894 |
| 8 | risk factors [Title/Abstract] OR dangerous factors [Title/Abstract] OR influencing factors [Title/Abstract] OR risks [Title/Abstract] OR Clinic Efficacy[Title/Abstract] OR Blood Press[Title/Abstract] OR Systolic[Title/Abstract] OR Diastolic [Title/Abstract] OR BP[Title/Abstract] OR Cholesterol [Title/Abstract] OR LDL[Title/Abstract] OR HDL[Title/Abstract] OR BMI [Title/Abstract] OR blood lipid [Title/Abstract] OR Waist circumference[Title/Abstract] OR Hip circumference[Title/Abstract] OR waist-hip ratio[Title/Abstract] OR Anxiety[Title/Abstract] OR Depressed[Title/Abstract] | 1636095 |
| 9 | #7 OR #8 | 3037525 |
| 10 | Randomized Controlled Trial [Publication Type] OR Controlled Clinical Trial[Publication Type] | 605069 |
| 11 | randomized controlled trials [Title/Abstract] OR clinical trials,randomize[Title/Abstract] OR trials, randomized clinical [Title/Abstract] OR randomized[Title/Abstract] OR randomised[Title/Abstract] OR randomly[Title/Abstract] OR trial[Title/Abstract] OR groups[Title/Abstract] | 2954356 |
| 12 | #10 OR #11 | 3101703 |
| 13 | #3 AND #6 AND #9AND #12 | 53 |

COCHRANE

| No. | Search Details | No. of Articles |
| --- | --- | --- |
| 1 | MeSH descriptor: [Coronary Artery Disease] explode all trees | 6373 |
| 2 | MeSH descriptor: [Arteriosclerosis] explode all trees | 10664 |
| 3 | MeSH descriptor: [Myocardial Ischemia] explode all trees | 28252 |
| 4 | MeSH descriptor: [Percutaneous Coronary Intervention] explode all trees | 5522 |
| 5 | MeSH descriptor: [Coronary Artery Bypass] explode all trees | 5416 |
| 6 | (“coronary artery disease” OR “coronary heart disease” OR (coronary NEXT isch*mia) OR (cardiac NEXT isch*mia) OR artheroscleros?s OR arterioscleros?s OR arterioloscleros?s OR “ischemic heart disease” OR “ischaemic heart disease” OR “ischemia heart disease” OR “ischaemia heart disease” OR (myocard* NEXT isch*mia) OR angina* OR (angina NEXT pectoris) OR (angor NEXT pectoris) OR (myocard* NEXT infarct*) OR “percutaneous coronary intervention” OR PCI OR “percutaneous transluminal coronary angioplasty” OR PTCA OR angioplast* OR stent* OR “coronary artery bypass grafting” OR CABG OR “coronary artery bypass” OR “acute coronary syndrome”):ti,ab,kw | 78960 |
| 7 | #1 OR #2 OR #3 OR #4 OR #5 OR #6 | 82575 |
| 8 | MeSH descriptor: [Risk Factors] explode all trees | 24286 |
| 9 | MeSH descriptor: [Epidemiologic Factors] explode all trees | 43949 |
| 10 | (“risk factor” OR “epidemiologic factor” OR “dangerous factor” OR “influencing factor” OR “clinic efficacy”) OR blood press OR BP OR systolic blood press OR SBP OR diastolic blood pressure OR DBP OR Cholesterol* OR TC OR low density lipoprotein OR LDL-c OR high density lipoprotein OR HDL-c OR (blood NEXT lipid*) OR BMI OR body mass index OR waist circumference OR hip circumference OR waist-hip circumference OR emotion* OR anxiety* OR depress*:ti,ab,kw | 269012 |
| 11 | #8 OR #9 OR #10 | 299442 |
| 12 | MeSH descriptor: [Telemedicine] explode all trees | 2524 |
| 13 | MeSH descriptor: [Mobile Health Units] explode all trees | 65 |
| 14 | MeSH descriptor: [Smartphone] explode all trees | 384 |
| 15 | MeSH descriptor: [Mobile Applications] explode all trees | 628 |
| 16 | MeSH descriptor: [Wearable Electronic Devices] explode all trees | 421 |
| 17 | (smartphone* OR cellphone* OR (smart NEXT phone*) OR (cell* NEXT phone*) OR (mobile NEXT phone*) OR (mobile NEXT technolog*) OR (mobile NEXT app*) OR (mobile NEXT health*) OR mhealth* OR m-health* OR (smartphone* NEXT app*) OR (phone NEXT app*) OR (cellphone NEXT app*) OR (cell-phone NEXT app*) OR WeChat* OR (WeChat* NEXT Message*) OR (text NEXT message*) OR text-based OR (Wearable* NEXT Devices*)OR telemedicine OR tele-medicine OR telehealth):ti,ab,kw | 15745 |
| 18 | #12 OR #13 OR #14 OR #15 OR #16 OR #17 | 53 |
| 19 | #7 AND #11 AND #18 | 250 |
| 20 | with Cochrane Library publication date Between Jan 1900 and May 2020, in Trials | 247 |

EMBASE

| No. | Search Details | No. of Articles |
| --- | --- | --- |
| 1 | 'coronary artery disease'/exp OR 'coronary artery disease' OR 'ischemic heart disease'/exp OR 'ischemic heart disease' OR 'arteriosclerosis'/exp OR 'arteriosclerosis' OR 'angina pectoris'/exp OR 'angina pectoris' OR 'heart infarction'/exp OR 'heart infarction' OR 'percutaneous coronary intervention'/exp OR 'percutaneous coronary intervention' OR 'angioplasty'/exp OR 'angioplasty' OR 'coronary artery bypass graft'/exp OR 'coronary artery bypass graft' OR 'coronary artery disease':ti,ab OR 'coronary heart disease':ti,ab OR 'coronary isch*mia':ti,ab OR 'cardiac isch*mia':ti,ab OR artheroscleros?s:ti,ab OR arterioscleros?s:ti,ab OR arterioloscleros?s:ti,ab OR 'isch*mic heart disease':ti,ab OR 'isch*mia heart disease' OR 'myocard* isch*mia':ti,ab OR angina*:ti,ab OR 'angor pectoris':ti,ab OR 'myocard* infarct*':ti,ab OR 'percutaneous coronary intervention':ti,ab OR pci:ti,ab OR 'percutaneous transluminal coronary angioplasty':ti,ab OR ptca:ti,ab OR angioplast*:ti,ab OR stent*:ti,ab OR 'coronary artery bypass grafting':ti,ab OR cabg:ti,ab OR 'coronary artery bypass':ti,ab OR 'acute coronary syndrome':ti,ab | 1297870 |
| 2 | 'risk factor*':ti,ab OR 'dangerous factor*':ti,ab OR 'influencing factor*':ti,ab OR 'risk*':ti,ab OR 'clinic efficacy':ti,ab OR 'blood press':ti,ab OR 'systolic':ti,ab OR 'diastolic':ti,ab OR 'bp*':ti,ab OR 'cholesterol':ti,ab OR 'ldl':ti,ab OR 'hdl':ti,ab OR 'bmi':ti,ab OR 'blood lipids':ti,ab OR 'waist circumference*':ti,ab OR 'hip circumference*':ti,ab OR 'waist-hip ratio':ti,ab OR 'anxiety':ti,ab OR 'depressed':ti,ab | 4533383 |
| 3 | trail*:de OR 'double-blind procedure':de OR 'randomized controlled trial':de OR 'single-blind procedure':de OR random*:de,ab,ti OR factorial*:de,ab,ti OR crossover*:de,ab,ti OR ((cross NEXT/1 over*):de,ab,ti) OR placebo*:de,ab,ti OR ((doubl* NEAR/1 blind*):de,ab,ti) OR ((singl* NEAR/1 blind*):de,ab,ti) OR assign*:de,ab,ti OR allocat*:de,ab,ti OR volunteer*:de,ab,ti | 2635717 |
| 4 | 'telemedicine'/exp OR 'text messaging'/exp OR 'mobile phone'/exp OR 'mobile application'/exp OR 'wearable device'/exp OR 'platform*':ti,ab OR 'smart phone*':ti,ab OR 'cell phone*':ti,ab OR 'mobile phone*':ti,ab OR 'mobile technolog*':ti,ab OR 'mobile app*':ti,ab OR 'smartphone app*':ti,ab OR 'smart-phone app*':ti,ab OR 'phone app*' OR 'cellphone app*' OR 'text messag*':ti,ab OR texting:ti,ab OR 'm health':ti,ab OR mhealth:ti,ab OR 'mobile health':ti,ab OR telemedicine:ti,ab OR 'tele medicine':ti,ab OR telehealth:ti,ab OR 'tele health':ti,ab OR wechat*:ti,ab OR 'wechat textmessag*':ti,ab OR 'wearable device*':ti,ab OR 'wearable electronic device*':ti,ab | 317122 |
| 5 | #1 AND #2 AND #3 AND #4 | 685 |

Web of Science

| No. | Search Details | No. of Articles |
| --- | --- | --- |
| 1 | TS=(coronary artery disease OR coronary heart disease OR CHD OR arteriosclerosis OR myocardial ischemia OR percutaneous coronary intervention OR coronary artery bypass OR coronary artery disease OR coronary OR cardiac OR ischemic heart disease OR ischaemic heart disease OR ischemia heart disease OR angina OR angina pectoris OR angor pectoris OR myocard infarct OR percutaneous coronary intervention OR PCI OR percutaneous transluminal coronary angioplasty OR PTCA OR angioplast OR stent OR coronary artery bypass grafting OR CABG OR coronary artery bypass OR acute coronary syndrome) | 360204 |
| 2 | TS=(risk factor OR epidemiologic factor OR dangerous factor OR influencing factor OR clinic efficacy OR blood press OR BP OR systolic blood press OR SBP OR diastolic blood pressure OR DBP OR Cholesterol OR TC OR low density lipoprotein OR LDL-c OR high density lipoprotein OR HDL-c OR blood lipid OR BMI OR body mass index OR waist circumference OR hip circumference OR waist-hip circumference OR emotion OR anxiety OR depress OR depressed OR depression） | 1149057 |
| 3 | TS=( smartphone OR cellphone OR mobile phone OR mobile technology OR application OR app OR mobile health OR mhealth OR m-health OR WeChat* OR WeChat message OR text OR text message OR text-based OR platform OR wearable device OR wearable electronic device OR telemedicine OR tele-medicine OR telehealth OR mobile health units) | 1598655 |
| 4 | TS=(trail OR double blind procedure OR randomized controlled OR single blind procedure OR random OR factorial OR crossover OR cross over OR placebo OR assign) | 701045 |
| 5 | #4 AND #3 AND #2 AND #1 | 515 |
